# Effect of Mouthwashes on Solubility and Sorption of Restorative Composites

**DOI:** 10.1155/2017/5865691

**Published:** 2017-06-08

**Authors:** José Pereira Leal, Jaqueline Damasceno da Silva, Rafaelle Fernanda Melão Leal, Carlos da Cunha Oliveira-Júnior, Vera Lúcia Gomes Prado, Glauber Campos Vale

**Affiliations:** ^1^Restorative Dentistry Department, Federal University of Piauí, Campus Universitário Ministro Petrônio Portella, SG10, 64049-550 Teresina, PI, Brazil; ^2^Superior Education Institute of Parnaiba Valley, 64212-790 Parnaiba, PI, Brazil

## Abstract

**Objective:**

Composites** s**orption and solubility can be precursors of several chemical and physical processes, which lead to deleterious effects on the polymer structure. This study evaluated the effect of mouthwashes on solubility and sorption of composite resins.

**Materials and Methods:**

Forty-two specimens of each evaluated composite (Filtek Bulk Fill Flow, Opallis Flow, Durafill VS, and Filtek Z350) were prepared and randomized into seven groups for each solution (mouth rinses with and without alcohol and distilled water) and stored for seven days. Solubility and sorption tests were performed according to ISO4049. Data were analyzed using 2-way-ANOVA followed by Tukey's test for means comparison (*α* = 0.05). In addition, paired *t*-test was performed to analyze the alcohol effect on the studied composite resin properties.

**Results:**

Listerine Cool Mint (containing alcohol in its composition) caused the greatest degree of sorption for all composites tested in comparison to other rinses, while for solubility this behavior was observed for Opallis Flow and Durafill VS composite resins (*p* < 0.05). Regarding the composites, Opallis Flow showed the highest sorption and solubility values in general (*p* < 0.05).

**Conclusion:**

Overall, the sorption and solubility of composites were higher in mouthwashes containing alcohol in its composition, with Opallis Flow being the most affected composite resin.

## 1. Introduction

Restorative composites are widely used in clinical dentistry because of their aesthetic quality and adhesion ability to dental structures. Their improvements in mechanical properties have led to a universal application and versatility, being indicated for both anterior and posterior teeth [1].

Even after an effective polymerization, composites exhibit instability and can interact with the environment. In the oral cavity, these materials are able to absorb water and to absorb and release chemical substances [2]. The phenomenon of sorption and solubility can be a precursor of several physical and chemical processes leading to deleterious effects on the structure and function of the polymeric material. These effects may include volumetric change as expansion, physical changes as plasticizing, and chemical changes such as oxidation and hydrolysis [3].

In this context, the resistance of material to the challenges in oral environment is essential to the longevity of adhesive restorations. The rate of sorption and solubility of these materials can be influenced by the individual composition of each material [4], the hydrophilic character of matrix, the degree of conversion, and the solvent [5–7]. Moreover, studies have reported that sorption and solubility are dependent on the immersion time [7] and pH of the solution [8].

In accordance with standard ISO 4049/2009, for a composite to be indicated as a restorative material, they must have water sorption lower than 40 mg/mm^3^ and lower solubility than 7.5 mg/mm^3^ within 7 days of storage. Therefore, apart from water, other solvents can result in deleterious effects on matrix composites [9, 10], since they are an intermittent or continuous source of chemical degradation [7, 11]. However, there are few studies designed to assess the influence of mouthwashes on the mechanical and chemical properties of these materials [10, 12], although there is a concern about their effects on the physical properties of composite resins such as discoloration, staining, and translucency [13].

In most cases, the formula of these mouth rinses contains water, antimicrobial agents, salts, preservatives, and alcohol in different concentrations [12]. However, the effects of these components in the polymer matrix of the composite resins are still the subject of much discussion [6]. Particularly, alcohol causes softening of the composite surface [14–16] by removing monomers, oligomers, and linear polymers of the formed polymer structure [17] or causes opening of the polymer structure, leading to decreased hardness and consequently increasing wear of the material [11, 16].

Thus, considering that the effect of mouthwashes on the properties of composite resins is still subject to debate, the aim of this study was to evaluate the effect of mouthwashes widely used on the solubility and sorption of composites with different compositions.

## 2. Materials and Methods

### 2.1. Restorative Materials and Solutions

Four types of composite resins were used: low shrinkage resin (Filtek Bulk Fill Flow), low viscosity resin (Opallis Flow), microparticulate (Durafill VS), and nanohybrid resin (Filtek Z350), whose basic description is in Table 1. The solutions used (Table 2) consisted of six types of mouthwashes routinely used for oral hygiene, three containing alcohol (Listerine Cool Mint, Plax Ice, and PerioGard) and three without alcohol (Listerine Zero, Plax Fresh Mint, and PerioGard without alcohol) and deionized distilled water as a control.

### 2.2. Preparation of Specimens

Forty-two specimens of each composite were randomly divided into seven groups (*n* = 6), totaling 168 units for the whole experiment. The specimens were obtained using a Teflon mold containing 6 circular perforations (4 mm diameter × 2 mm thick). The composite resin insertion inside the Teflon matrix was performed in a single increment. A polyester strip was placed and a glass slide (weighing 272 g) was pressed for 10 s against the material for removing excess and the surface of each specimen to acquire a smooth and flat appearance. After, 40 s of photoactivation was applied according to the manufacturer's specifications. After this time, the specimens were removed from the matrix and placed in labeled test tubes. The composites were light-polymerized using a halogen-based light-curing unit (Optilux 400, Demetron Research Corporation, Danbury, CT, USA). The light output was tested (480 ± 32 mW/cm^2^) before each use with a Demetron Model 100 radiometer (Demetron Research Corporation, Danbury, CT, USA).

Subsequently, the samples were polished with sandpaper discs (soft flex TDV) under low speed to remove the excess and the debris was removed with a light jet of air.

### 2.3. Sorption and Solubility Measurements

The measurement of the composite resins sorption and solubility was performed in accordance with ISO 4049. The test specimens were stored in a desiccator with blue silica gel and after 24 hours were weighed on an analytical balance to obtain a stable initial weight. This cycle was repeated 24 hours until a constant mass (*m*1) was observed. After stabilization of the initial mass, diameter and thickness of the specimens were measured using a digital caliper (±0.001 mm). The diameter of each sample was measured at two points perpendicular to one another and the average diameter was calculated. The thickness of each specimen was measured at the center in four equally spaced points and average thickness was calculated. To calculate the volume (*V*) of the specimen, the following formula was used: *V* = *π* × *r*2 × *h*, where *r* is the radius of the average (diameter/2) and *h* is the average thickness.

After determining the volume of the test specimens, they were stored separately in 2 mL of each solution for seven days, with the solution being changed daily. After this period, the samples were removed with tweezers, abundantly washed with distilled water and dried with an absorbent paper towel, kept at room temperature for 15 s, and reweighed to obtain the mass after immersion in solutions (*m*2). The specimens were then replaced again in their tubes and stored in a desiccator with silica gel. Measurements during dehydration were performed again using the same methodology described in cycles of 24 hours to obtain the reconditioned constant mass, called “*m*3.”

The average sorption and solubility (mg/mm^3^) of each specimen were calculated according to the following equations: Sorption = *m*2 − *m*3/*V*; Solubility = *m*1 − *m*3/*V*, where *m*1 is mass after initial drying specimen (ug), *m*2 is mass after the immersion period in solutions (ug), *m*3 is final mass after drying (ug), and *V* is volume in mm^3^.

### 2.4. Statistical Analysis

All data have normal distribution of errors and were analyzed by 2-way-ANOVA, considering the composite resins and mouthwashes as the main factors under study. Post hoc Tukey test was used to compare means of sorption and solubility in studied factors. To evaluate the alcohol effect on sorption and solubility, data were grouped and paired by mouth rinses with alcohol and without alcohol and paired *t*-test was performed. The SAS program version 9.0 was used to perform statistical tests with significance level set at 5%.

## 3. Results

The 2-way-ANOVA showed significant effects for composite resins, mouthwashes, and their interaction for both sorption and solubility results (*p* < 0.0001). The results of the sorption test are shown in Table 3. With respect to sorption caused by rinses, for all studied composite resins, Listerine with alcohol (Listerine Cool Mint) caused a greater extent of sorption, being superior to all other mouthwashes with alcohol tested (*p* < 0.05). Overall, Opallis Flow showed the higher values of sorption with statistical difference compared to the other composite resins, when Listerine with alcohol, PerioGard without alcohol, and water were used (*p* < 0.05).

The results of the solubility test are described in Table 4. For Opallis Flow, Listerine with alcohol led to increased solubility of the composite in comparison to other rinses (*p* < 0.05) which did not differ between them. Filtek 350 showed higher solubility values for Listerine, PerioGard, and Plax with alcohol rinses, which did not differ between them (*p* > 0.05). In general, Opallis Flow showed the highest solubility values in all tested solutions (*p* < 0.05), except for Plax and PerioGard with alcohol. In the first, the solubility values did not differ between the composites (*p* > 0.05), while, in the second, there was no statistically significant difference between Opallis Flow, Durafill VS, and Filtek Bulk Fill (*p* > 0.05). Furthermore, Filtek Z350, Durafill VS, and Filtek Bulk Fill also behaved similarly when this rinse was used.

**Figure 1:**
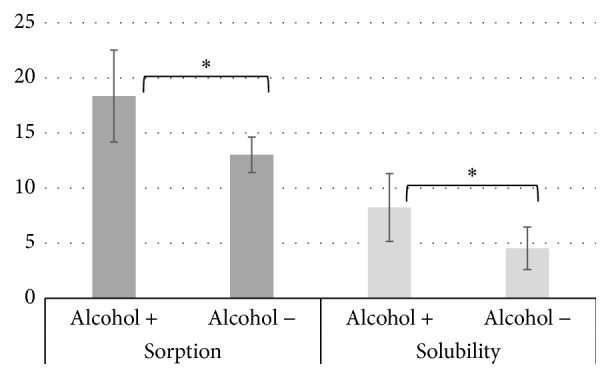
Sorption and solubility (mean ± SD, *μ*g/mm^3^) of composite resins in the mouthwashes according to the presence or absence of alcohol (*n* = 36). The asterisk indicates a significant difference (*p* < 0.05).

It is clearly observed that the mouthwashes containing alcohol in the composition led to higher values of sorption and solubility, as may be seen in Figure 1, where data of all composite resins were grouped and paired with respect to presence or absence of alcohol in mouthwash.

## 4. Discussion

Sorption is a diffusion-controlled process that occurs in the organic fraction of composite resin and seems to be related to its potential hydrophilicity and chemical composition of the filler particles. Thus, the kinetics of this process can be slower or faster for some composites, depending on its composition [18]. Triethylene glycol dimethacrylate (TEGDMA), a monomer present in the studied composites, is the one with the greater hydrophilicity and greater sorption capacity [18]. Among the composite resins analyzed in this study, Opallis Flow was the one with the worst performance, in which a greater degree of sorption and solubility was observed in comparison to other composites. This behavior can be explained by TEGDMA presence in their chemical composition. The other composite resins, which also had TEGDMA, differs from Opallis Flow because they have other monomers in their compositions such as UDMA or Bis-GMA, which are less hydrophilic than TEGDMA.

Similarly, the high sorption of Bis-GMA and TEGDMA is due to the hydroxyl groups and ether linkages, respectively, in these monomers. The UDMA has less solvent sorption than those due to the presence of urethane groups [19]. Khokhar et al. [20] observed that, in normal conditions, the UDMA showed lower water sorption than Bis-GMA, which corroborates this research, since the composite resins containing UDMA in its composition (Filtek Bulk Fill, Durafill VS, and Filtek Z350) showed lower sorption values.

On the other hand, solubility is a measure of the amount of residual unconverted monomer which is released in the solution and may have the potential to impact on the stability of the material structure [18]. The composite solubility is related to the sorption of the same composite since the solvent must penetrate into the polymer, so that leachable components could be released to the material outside [21], which is in agreement with this study (Tables 3 and 4). However, other factors such as the degree of conversion and the crosslinked net density may have greater importance in the correlation sorption/solubility [22]. It is known that hydrophilic materials showed enhanced degradation by sorption and solubility in water than hydrophobic materials; however hydrophobic monomers such as Bis-GMA and UDMA, in the composition of three tested composite resins (Filtek Bulk Fill, Durafill VS, and Filtek Z350), are also susceptible to chemical reactions by alcohol [22].

The alcohol is used in mouthwashes as a solvent, flavor enhancer, and antiseptic agent [23]. In general, it was found that mouthwashes containing alcohol in their composition showed higher sorption and solubility in the evaluated composites (Figure 1), especially “Listerine with alcohol” which contains the highest alcohol concentration (approximately 30%). This can be explained because ethanol penetrates the polymer network causing expansion of the polymer structure, allowing the release of residual monomers and causing dissolution of the linear polymer chain [24].

According to the ISO 4049 (2000), in order for composites to be indicated as restorative materials, they must have water sorption lower than 40 g/mm^3^ and solubility lower than 7.5 mg/mm^3^ for a period of 7 days of storage. The sorption values of all composite resins were lower than the recommended values while regarding solubility, some composite resins had higher values than recommended, especially in solutions containing alcohol in their composition (Tables 3 and 4). This research has the limitation of an in vitro assay; therefore, the results should be carefully interpreted. Clinical studies must be conducted to confirm the results.

## 5. Conclusion

It can be concluded that the sorption and solubility of tested composites were higher in mouthwashes containing alcohol. Thus, mouthwashes without alcohol should be preferred in patients with extensive restorations. The composite resins that had worst and better performance regarding the properties studied were Opallis Flow and Filtek Z350, respectively.

## Figures and Tables

**Table 1:** Restorative materials used according to classification, manufacturers, and composition.

Material	Manufacturer	Inorganic contents	Organic matrix	Classification
Bulk Fill Flow	3M ESPE (St. Paul, EUA)	Zirconia/silica ytterbium trifluoride	BIS-GMA, UDMA, BIS-EMA and Procylat.	Low shrinkage resin

Opallis Flow	FGM (Joinville, Brazil)	Aluminum silicate barium glass	BIS-GMA, BIS-EMA, TEG DMA.	Low viscosity resin

Durafill VS	Heraeus Kulzer (Hanau, Germany)	Silicon dioxide and prepolymerized particle	BIS-GMA, TEG-DMA, and UDMA.	Microparticulate composite resin

Z-350	3M ESPE (St. Paul, EUA)	Zirconia/silica	BIS-GMA, TEG-DMA, UDMA, BIS-EMA, Dimethacrylate, polyethylene glycol, BHT, and pigments.	Nanohybrid composite resin

**Table 2:** Composition of Mouthwashes used in this study.

Mouthwashes	Manufacturer	Composition
Listerine zero	Johnson & Johnson Healthcare Prod.	Thymol, eucalyptol, methyl salicylate, menthol, water, sorbitol solution, poloxamer 407, benzoic acid, mint and peppermint essences, sodium saccharin, sodium benzoate, green dye 3.

Listerine Cool Mint	Johnson & Johnson Healthcare Prod.	Thymol, eucalyptol, methyl salicylate, menthol, water, sorbitol solution, alcohol (30%), poloxamer 407, benzoic acid, mint and mint essences, sodium saccharin, sodium benzoate, green dye 3.

Colgate Plax Fresh Mint	Colgate-Palmolive Ind. Com. Ltda.	Sodium fluoride, cetylpyridinium chloride, water, glycerin, propylene glycol, sorbitol, poloxamer 407, sodium chloride, potassium sorbate, sodium saccharin, citric acid, green dye, yellow dye.

Colgate Plax Ice	Colgate-Palmolive Ind. Com. Ltda.	Sodium fluoride, cetylpyridinium chloride, water, glycerin, propylene glycol, 21.6% alcohol, sorbitol, poloxamer 338, poloxamer 407, potassium sorbate, sodium saccharin, citric acid, sucralose, blue dye

PerioGard with alcohol	Colgate-Palmolive Ind. Com. Ltda.	Chlorhexidine gluconate 0.12%, water, glycerin, ethanol, polysorbate 20, mint flavor aromatic composition, sodium saccharinate, FD & C, Blue 1.

PerioGard without alcohol	Colgate-Palmolive Ind. Com. Ltda.	Chlorhexidine gluconate, water, glycerin, polysorbate 20, mint flavor aromatic composition, sodium saccharinate, FD & C, Blue 1.

Water d.d.	—	—

**Table 3:** Average (±SD) of sorption (*μ*g/mm^3^) according to composite resin and mouthwashes (*n* = 6).

Mouthwashes	Composites resins			
	Filtek Bulk Fill Flow	Opallis Flow	Durafill VS	Filtek Z350 XT
Listerine with alcohol	21,65 (1,48) B,a	28,52 (1,69) A,a	22,22 (1,49) B,a	20,16 (0,64) B,a
Listerine without alcohol	13,46 (0,99) A,cd	15,13 (1,49) A,cd	14,49 (1,70) A,bc	13,12 (1,39) A,cd
PerioGard with alcohol	15,68 (1,33) AB,b	17,92 (1,30) A,b	16,12 (1,45) AB,b	15,34 (1,54) B,b
PerioGard without alcohol	11,96 (1,14) B,de	14,78 (1,12) A,d	12,08 (1,08) B,d	11,83 (0,84) B,de
Plax with alcohol	14,69 (1,61) B,bc	17,17 (0,88) A,bc	16,39 (1,32) AB,b	14,42 (0,92) B,bc
Plax without alcohol	11,70 (0,61) B,de	13,68 (1,21) A,d	12,38 (0,88) AB,cd	11,71 (0,90) B,de
Water	11,36 (0,45) B,e	14,31 (0,95) A,d	11,85 (0,95) B,d	11,23 (0,46) B,e

Different lowercase letters indicate statistical difference in columns and different uppercase letters indicate statistical difference in rows (*p* < 0.05).

**Table 4:** Average (±SD) of solubility (*μ*g/mm^3^) according to composite resin and mouthwashes (*n* = 6).

Mouthwashes	Composites resins			
	Filtek Bulk Fill Flow	Opallis Flow	Durafill VS	Filtek Z350 XT
Listerine with alcohol	8,48 (1,12) C,a	15,30 (2,14) A,a	12,34 (1,37) B,a	6,50 (0,74) C,a
Listerine without alcohol	4,82 (0,83) B,c	7,71 (1,64) A,b	4,55 (1,53) B,cd	3,76 (1,26) B,bc
PerioGard with alcohol	6,80 (0,88) AB,ab	8,51 (1,10) A,b	6,77 (1,80) AB,bc	5,35 (1,42) B,ab
PerioGard without alcohol	4,09 (0,87) B,c	6,42 (1,72) A,b	3,66 (1,55) B,d	2,63 (0,82) B,c
Plax with alcohol	6,64 (1,37) A,b	8,44 (1,56) A,b	7,85 (1,63) A,b	6,00 (1,86) A,a
Plax without alcohol	3,88 (0,79) B,c	6,60 (1,45) A,b	3,50 (1,46) B,d	2,88 (0,85) B,c
Water	3,72 (0,70) B,c	6,34 (1,41) A,b	3,86 (1,36) B,d	2,08 (0,93) B,c

Different lowercase letters indicate statistical difference in columns and different uppercase letters indicate statistical difference in rows (*p* < 0.05).
